# California State Dental Association

**Published:** 1873-08

**Authors:** H. J. Plomteaux

**Affiliations:** Secretary.


### AMERICAN JOURNAL

OF

### DENTAL SCIENCE.

Vol. VII. THIRD SERIES--AUGUST, 1873. No. 4.

### ARTICLE I.

### *California State Dental Association.*

Pursuant to adjournment, the fourth annual session of the California State Dental Association convened at St. Andrew's Hall, Young Men's Christian Association Building, San Francisco, May 20th, 1873, at 10 o'clock A. M., and was called to order by the President, Wm. Dutch, D. D. S.

Prayer by Rev. Dr. Cox, of San Francisco.

Officers all present except Vice-President S. M. Harris, who was absent on account of illness.

*New Members.*—During the session which continued four days, the following named gentlemen were admitted to active membership:

J. W. Winter, Alex. Warner, Jr., D. D. S., C. W. Richards, D. J. Nis, W. H. Stanley, J. B. Beers, T. H. Furgerson, D. D. S., C. H. Fuller and A. B. Wood, of San Francisco; G. C. Hoadley, of San Jose; F. H. Hubbard, M. D., of Sacramento; J. J. Dyar, of Santa Cruz; D. W. Powers, of Oakland; and A. Cornwall, of Virginia City, Nevada.

*Honorary Members.*—Dr. O. D. Munson, E. S. Belden, M. D., of San Francisco; and B. S. Codman, of Boston, Massachusetts.

Essays were read as follows:

Requisites of the Dentist. By W. J. Prather.

Circulation of the Blood. By H. Austin.

The Mouth, its Structure and Function. By R. Cutlar.

Operative Dentistry. By J. W. Myres.

Causes and Treatment of Offensive Breath. By Max Sichel.

Dental Ethics. By F. E. Bunnell.

*Reports of Standing Committees.*—Reports were received from Drs. Menefee, Bush and McCowen, of Committee on Mechanical Dentistry; Dennis, on Operative Dentistry; and S. E. Knowles, on Dental Pathology and Surgery.

*Special Committees.*—The Legislative Committee recommended a bill similar to the Ohio law, giving members of the profession, now residents of the State, two years to prepare for an examination. Report adopted and committee instructed to use all legitimate means to secure its passage at next session of the Legislature.

The Revision Committee reported a revised Constitution, By-Laws, Code of Ethics, etc., which were adopted. The Code of Ethics is the same as that of the American Dental Association.

The Barnum Testimonial Committee accompanied their report with a magnificent testimonial, which will be presented to Dr. Barnum at the next session of the American Dental Association; therefore I refrain from giving a description of it here: although as marked by the committee, "it is a paltry gift to one who has proven himself a public benefactor by bestowing such a noble *free gift* upon the profession, when he had a fortune within his reach had he patented his invention. Paltry though it be, it is one that he will be proud of, and it will reflect credit upon our Association."

*Dental College.*—President Dutch, in his annual address, said, "It is evident to us all that we need on the Pacific coast a school of instruction for those who would qualify themselves for the practice of dentistry; a school where the science and the art, the theory and the practice of dentistry may be acquired, and the power of publishing to the world, as an authority to be respected, the proficiency of those who have been thoroughly taught, as by the giving of diplomas." He also recommended the selection of eight from our number, who might be deemed the best qualified, and who were willing to take upon themselves such a responsibility, requesting them to organize from among themselves, as far as they can, a dental college, affiliating with the University of California, or some other city organization, for the purpose of conferring the degree of D. D. S.; or, failing in such satisfactory affiliation, then asking the Legislature to confer the power on the college itself; and that you give such an organization all the support you can, individually and collectively."

Prof. Gillman, President of the University of California, having been invited, addressed the Association on the above subject. He spoke of the University as an "institution of learning established by the State on foundations which were laid by private devotions in the days or Argonauts, who believed in the wisdom which was better than gold. It has been endowed by the National Government with magnificent land grants, and received from the State, generous donations and a comprehensive charter. It is a university of the nineteenth century, open without money and without price to students of every State and country, designed to promote every branch of liberal culture." He would be glad if in the organization of a dental school in connection with the University, they could rely upon the co-operation of the California State Dental Association; all the authorities of the University would rejoice at suggestions and help and counsel from members of this Society.

The Professor's address was received with applause, and he concluded by saying that he had "always dreaded meeting a single gentleman of our profession, and dreaded much more coming into the hands of thirty or forty;" but hoped from the reception given him, that he would be able to report that we were united, and would be found co-operating with the University in the interests of science and humanity.

A committee of five was appointed to confer with the Board of Regents relative to the matter, and further consideration of it postponed until 10.30 o'clock next day, when the committee reported the following resolutions, which were unanimously adopted.

*Resolved*, that this Association receives with unqualified satisfaction the assurance that our most cherished hopes are to be realized through the wisdom and liberality of the Board of Regents of the University of California, in their proposed establishment of a dental college.

*Resolved*, that we approve of and recommend the following *curriculum* of instruction in the college:—1. General Anatomy and Surgery. 2. Physiology. 3. Materia Medica and Therapeutics. 4. Chemistry and Toxicology. 5. Special Anatomy and Surgery. 6. Operative Dentistry and Pathology. 7. Dental Prosthesis and Metallurgy. 8. Institutes of Dental Science. 9. Demonstrators of Operative Dentistry. 10. Demonstrators of Dental Prosthesis. 11. Course of Clinical Lectures. 12. Board of Examiners.

*Resolved*, that the Association endorse and recommend the following named gentlemen as proper persons to fill the special chairs above designated:—Drs. Wm. Dutch, S. W. Dennis, C. C. Knowles, Wm. J. Younger, H. E. Knox, S. E. Knowles, H. Austin and J. J. Menefee.

*Resolved*, that an Advisory Committee of five be appointed and empowered to act with the Advisory Committee of the Board of Regents in carrying into effect all necessary details of organization.

The president appointed Drs. Cutlar, Birge, Lundbory, Bunnell and Coyswell, to act as Advisory Committee.

Clinics were given during the session by Drs. Dutch, Dennis, Knowles and Younger.

*Discussions.*—The several reports and essays were thoroughly discussed, and as soon as published, each dental journal will be furnished a copy, so that they can select therefrom any matter that they may deem new or important to their readers and the profession generally.

*Officers.*—President, Dr. C. C. Knowles, San Francisco; Vice-President, Dr. J. J. Menefee, San Jose; Secretary, Dr. H. J. Plomteaux, Woodland; Corresponding Secretary, Dr. H. E. Knox, San Francisco; Treasurer, H. Austin, San Francisco; Librarian, J. J. Birge, San Francisco.

Standing Committees were appointed on Dental Pathology and Surgery, Dental Therapeutics, Dental Chemistry, Operative Dentistry, Mechanical Dentistry, Dental Histology and Microscopy, Dental Literature and Education, Publication, and Executive.

*Theses.*—The President made the following assignments: An Inquiry into the Causes and Treatment of Degeneracy of Children's Teeth, Dr. D. M. Hughes. The Secretions of the Mouth, Considered in their Normal and Abnormal Characteristics, Dr. T. H. Ferguson. Pregnancy as Affecting the Teeth, Dr. A. Cornwall. The Inter-relations of Dentistry and Medicine, Dr. J. A. W. Lundbory. Extraction of Teeth, Dr. F. M. Shields. Dental Education, Dr. H. J. Plomteaux.

*The next annual session* will be held in San Francisco, commencing second Tuesday in June, 1874, at 10 A. M.

Resolutions appropriate to the memory of Dr. E. O. Belle, deceased since our last annual session, were reported and adopted by standing vote.

Mr. F. P. Forbes, of San Francisco, presented the Association, for the President's use, an elegant marble slab and gavel, which was received with applause, and a vote of thanks extended the donor.

The able and efficient administration of President Dutch was highly endorsed by the Association.

The session lasted four days, and with short intermissions, was at work from 10 o'clock A. M. to 10 o'clock P. M.

After adjournment we proceeded to Martin's restaurant and partook of an excellent collation given us by the San Francisco Dental Association.

H. J. Plomteaux, *Secretary.*

